# Effects of Aurora kinase A on mouse decidualization via Stat3-plk1-cdk1 pathway

**DOI:** 10.1186/s12958-021-00847-5

**Published:** 2021-10-29

**Authors:** Peng-Chao Wang, Si-Ting Chen, Zeng-Ming Yang

**Affiliations:** 1 College of Veterinary Medicine, Shanxi Agricultural University, Taigu, 030801 China; 2 College of Veterinary Medicine, South China Agricultural University, Guangzhou, 510642 China

**Keywords:** Uterus, Decidualization, Aurora A, Stat3, Plk1, Cdk1

## Abstract

**Background:**

Decidualization is essential to the successful pregnancy in mice. The molecular mechanisms and effects of Aurora kinase A (Aurora A) remain poorly understood during pregnancy. This study is the first to investigate the expression and role of Aurora A during mouse decidualization.

**Methods:**

Quantitative real time polymerase chain reaction, western blotting and in situ hybridization were used to determine the expression of Aurora A in mouse uteri. Aurora A activity was inhibited by Aurora A inhibitor to explore the role of Aurora A on decidualization via regulating the Aurora A/Stat3/Plk1/Cdk1 signaling pathway.

**Results:**

Aurora A was strongly expressed at implantation sites compared with inter-implantation sites. Furthermore, Aurora A was also significantly increased in oil-induced deciduoma compared with control. Both Aurora A mRNA and protein were significantly increased under in vitro decidualization. Under in vitro decidualization, Prl8a2, a marker of mouse decidualization, was significantly decreased by TC-S 7010, an Aurora A inhibitor. Additionally, Prl8a2 was reduced by Stat3 inhibitor, Plk1 inhibitor and Cdk1 inhibitor, respectively. Moreover, the protein levels of p-Stat3, p-Plk1 and p-Cdk1 were suppressed by TC-S 7010. The protein levels of p-Stat3, p-Plk1 and p-Cdk1 were also suppressed by S3I-201, a Stat3 inhibitor). SBE 13 HCl (Plk1 inhibitor) could reduce the protein levels of p-Plk1 and p-Cdk1. Collectively, Aurora A could regulate Stat3/Plk1/Cdk1 signaling pathway.

**Conclusion:**

Our study shows that Aurora A is expressed in decidual cells and should be important for mouse decidualization. Aurora A/Stat3/Plk1/Cdk1 signaling pathway may be involved in mouse decidualization.

## Introduction

Embryo implantation is an interaction between receptive endometrium and competent blastocysts, and is essential for achieving successful pregnancy. In rodents and humans, decidualization occurs during early pregnancy. During decidualization, the fibroblast-like endometrial stromal cells transiently proliferate and differentiate into large, epithelial decidual cells [1, 2]. Although rodent decidualization requires the stimulus from embryos, decidualization can also be induced by artificial stimuli [2, 3]. Different from most mammals, human decidualization does not require embryo implantation and spontaneously occur during the mid-secretory phase of the menstrual cycle [4]. Accumulating evidences suggest that defective decidualization is associated with preeclampsia [5]. However, the molecular mechanism underlying decidualization remains poorly defined.

Aurora kinases include Aurora A, Aurora B and Aurora C, and are involved in cell division [6]. The expression of Aurora A fluctuates during the cell cycle, being inactivated in the G1 phase, and quickly rising in G2 and peaking at early mitosis [7]. Aurora A is localizes to the centrosomes in G2/M phases and is also present on the mitotic spindle in M phase [8]. Aurora A is a serine/threonine kinase and participates in centrosome duplication to control mitosis [9]. Aurora A could promote centrosome maturation and bipolar spindle assembly [8]. Growth hormone enhances Aurora A expression during maturation of human oocytes in vitro [10]. Aurora A knock-out mice die early during embryonic development before the 16-cell stage and have defects in mitosis [11].

Stat3 phosphorylation is detected in luminal epithelium during mouse embryo implantation [12]. Deletion or local inhibition of Stat3 results in implantation failure [13–15]. Aurora A knockdown or inhibition can notably decrease Stat3 phosphorylation [16]. Stat3 can regulate Polo-like kinase 1(Plk1) to participate in centrosomes clustering [17]. Plk1 is a serine/threonine kinase and regulates cell division [18, 19]. Plk1 is phosphorylated and activated by Aurora A to promote spindle formation during mitosis [18–20]. Moreover, Plk1 plays a crucial role in activation of cyclin B-Cdk1 complexes [21]. Cdk1, also known as cell division control protein 2 (Cdc2), is activated by other mitotic protein kinases to control the onset of mitotic phase [22, 23]. Cdk1 expression is gradually reduced from days 5 to 8 in pregnant mouse uterus [24].

This study was to explore the regulation and role of Aurora A during mouse decidualization. We demonstrated that Aurora A is highly expressed in decidual cells. Inhibition of Aurora A would dampen mouse decidualization in vivo and vitro. Our data showed that Aurora A/Stat3/Plk1/Cdk1 signaling pathway was very important during decidualization.

## Materials and methods

### Animals and treatments

Adult CD1 mice were maintained in a temperature- and light-controlled SPF fertility (14 h light: 10 h dark cycle). All mouse protocols were approved by Institutional Animal Care and Use Committee of South China Agricultural University. Female mice were mated with fertile or vasectomized males to induce pseudopregnancy or pregnancy (day 1 is the day of vaginal plug). From days 1 to 4, pregnancy was confirmed by flushing embryos from oviducts or uteri. The implantation sites on day 5 were visualized through intravenous injection of 100 μl of 1% Chicago blue dye (Sigma Aldrich Inc., St. Louis, USA) in saline. Delayed implantation and artificial decidualization were performed as previously described [25].

Pregnant or pseudopregnant mice were intraperitoneally injected with 30 mg/kg TC-S 7010 (a specific inhibitor of Aurora A, S1451, Selleck, 60 mg/ml dissolved in DMSO and diluted in saline, 100 μl per mouse,) on days 6 and 7. Injection of an equal volume of solvent was served as a control. On day 8, uteri were collected to calculate the weights of implantation sites and deciduoma. The dosage of TC-S 7010 was chosen as previously described [26].

### Isolation and treatments of primary uterine stromal cells

Primary uterine stromal cells were isolated as previously described [25]. In brief, the stromal cells were isolated from mouse uteri on day 4 of pregnancy with HBSS containing 1% (w/v) trypsin (Amresco, Solon, OH) and 6 mg/ml dispase (Roche Applied Science). Cells were cultured with DMEM/F12 (Sigma) containing 10% charcoal-treated FBS (Biological Industries, Israel) in 12-well or 6-well culture plates. After an initial culture for 2–3 h, cells were overnight starved for synchronization in the absence of FBS [27].

In vitro decidualization was induced as previously described [24]. Briefly, stromal cells were induced for decidualization with 1 μM progesterone (Sigma) plus 10 nM estradiol-17β (Sigma) in DMEM/F-12 medium containing 2% charcoal-stripped-FBS. For further studies, stromal cells were treated with TC-S 7010, S3I-201 (Stat3 inhibitor, 573,102, Sigma), SBE 13 HCl (Plk1 inhibitor, S7720, Selleck), or RO-3306 (Cdk1 inhibitor, S7747, Selleck).

### In situ hybridization

In situ hybridization was performed as previously described [24]. Total RNAs were extracted from pregnant uterus on day 5 of pregnancy and reverse-transcribed into cDNA for amplifying hybridization probe template with the corresponding primers (Table 1). The amplified fragment of Aurora A was cloned into pGEM-T vector plasmid (Promega, Madison, WI) and verified by sequencing. Digoxigenin-labeled antisense or sense cRNA probes were transcribed in vitro by digoxigenin RNA labeling kit (Roche Applied Science). Frozen sections (10 μm) were mounted on 3-aminopropyltriethooxysilane (Sigma)-treated slides and fixed in 4% paraformaldehyde solution in PBS. The sections were hybridized with digoxigenin-labeled probes at 55 °C for 16 h and incubated with sheep anti-digoxigenin antibody conjugated to alkaline phosphatase (1:5000, Roche). The signal was visualized with the buffer containing 0.4 mM 5-bromo-4-chloro-3-indolyl phosphate and 0.4 mM nitrobluetetrazolium. Endogenous alkaline phosphatase activity was inhibited with 2 mM levamisole (Sigma). The sections were counterstained with 1% methyl green. The positive signal of in situ hybridization was visualized as a dark brown color.

**Table 1:** Primers used in this study

Gene	Primer sequences	Accession number	Size (bp)	Application
Aurora A	CGTCTTGGTGACTGAGCAGATT	NM_001291185.1	500	In situ hybridization
	AGCCATACAGCCTGAGGATGT			
Aurora A	TCCTGGCTCTGAAGGTGCTGTT	NM_001291185.1	145	Real-time PCR
	ACTCGGGTGGCGTCATGGAAA			
Prl8a2	AGCCAGAAATCACTGCCACT	NM_010088	119	Real-time PCR
	TGATCCATGCACCCATAAAA			
Rpl7	GCAGATGTACCGCACTGAGATTC	M29016	129	Real-time PCR
	ACCTTTGGGCTTACTCCATTGATA			

### RNA extraction and quantitative real-time PCR

Cultured cells were collected for extracting total RNAs with Trizol (TaKaRa, Dalian, China). The RNAs (62.5 ng/μl) were then reverse transcribed into cDNAs with the PrimeScript reverse transcriptase reagent kit (TaKaRa, Tokyo, Japan). PCR conditions used for cDNA template synthesis were 15 min at 37 °C, 30 s at 85 °C and saved at 4 °C. The cDNAs were amplified by SYBR Premix Ex Taq kit (TaKaRa; DRR041S) on the BIORAD-CFX96 TM Real-Time System (BioRad). The sequences of primers used for real-time PCR were listed in Table 1. The ΔΔCt method was employed to determine relative changes of gene expression compared to RPL7.

### Western blot

Western blot was performed as previously described [24] . The protein lysates of uterine tissues or cells were separated by SDS-polyacrylamide gel electrophoresis and transferred to a polyvinylidene fluoride membrane. After blocked in nonfat milk, membranes were incubated 16 h at 4 °C with each primary antibody for anti-Aurora A (ab108353, Abcam), anti-PLK1 (phospho T210) (ab155095, Abcam), anti-Tubulin (2144, CST), anti-β-actin (4970, CST), anti-P-STAT3 (9145, CST) or anti-Phospho-cdc2 (Tyr15) (4539, CST), respectively. Membranes were incubated in matched second antibody for 1 h. The ECL chemiluminescent kit (Amersham Biosciences) was used to visualize signals.

### Statistical analysis

All the data shown were the representatives from at least three independent experiments. Data were analyzed with Student’s t-test for comparing two groups or One-way ANOVA test for multiple groups. *P* < 0.05 was defined as a statistically significant.

## Results

### Expression of Aurora A expression in mouse uterus during early pregnancy

**Fig. 1:**
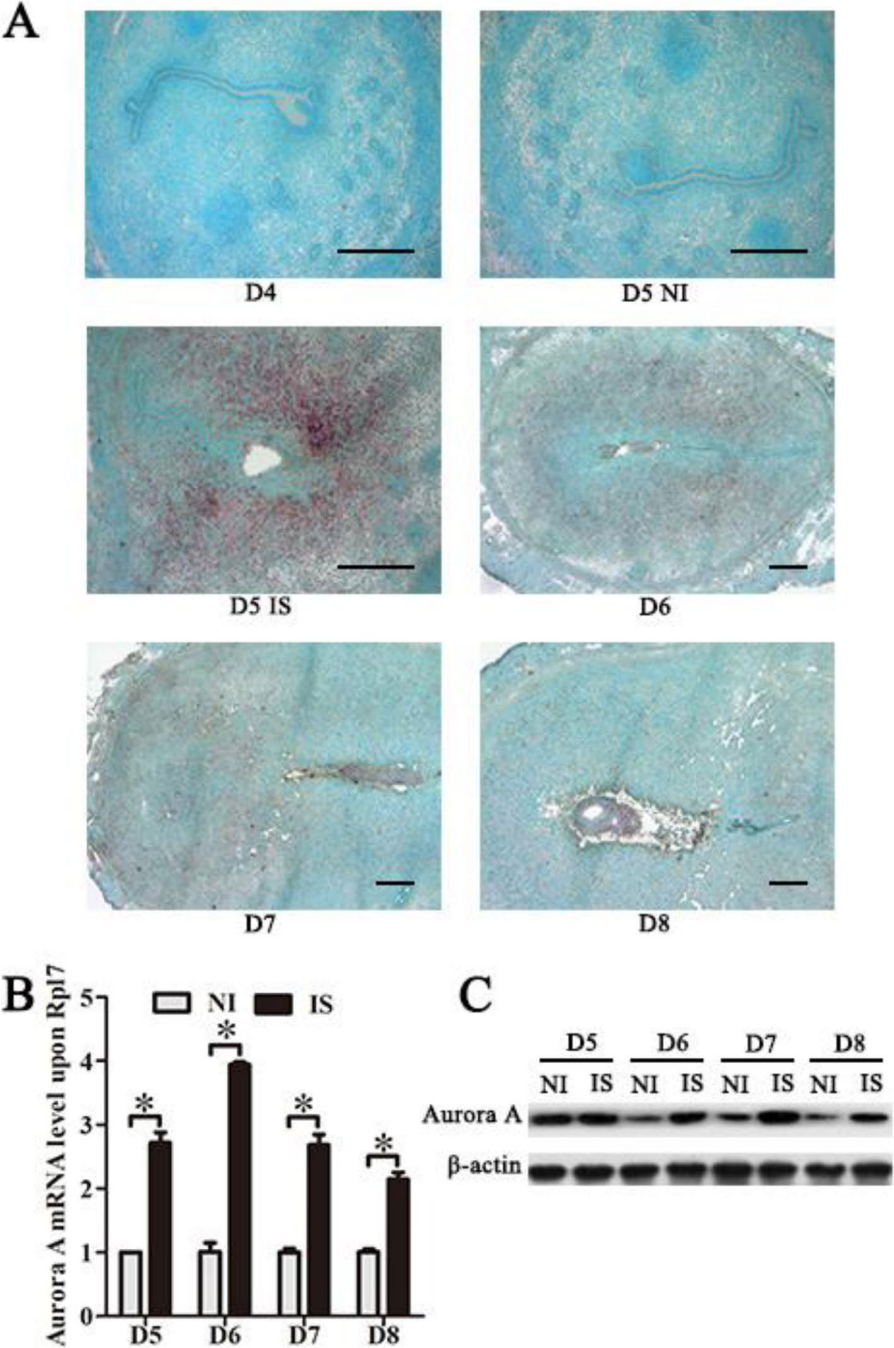
Aurora A expression in mouse uterus during early pregnancy. **A** In situ hybridization of Aurora A mRNA in mouse uteri from days 4 to 8. **B** Real-time PCR analysis of the mRNA level of Aurora A in mouse uterus from days 5 to 8 (NI, inter-implantation site; IS, implantation sites). **C** Western blot of Aurora A protein in mouse uteri from days 5 to 8. β-actin was used as a loading control. Data are presented as the mean ± SD, *p < 0.05, bar, 300 μm

In situ hybridization was performed to examine the spatial distribution of Aurora A mRNA in mouse uterus. There was no visible Aurora A mRNA signal in the uteri from days 1 to 4 of pregnancy (Fig. 1A). On day 5 of pregnancy, Aurora A mRNA signal was obviously detected in the subluminal stroma at implanting sites, whereas there was no detectable expression of Aurora A at the inter-implantation sites (Fig. 1A). From days 6 to 8, Aurora A expression was weakly detected in decidual cells (Fig. 1A). To further quantify the expression level of Aurora A by real-time RT-PCR, Aurora A mRNA level was strongly upregulated at implantation sites from days 5 to 8 compared with inter-implantation sites (Fig. 1B). Furthermore, Aurora A protein level was significantly increased at implantation sites from days 6 to 8 by Western blot compared to inter-implantation sites (Fig. 1C).

### Aurora A expression under delayed and activated implantation

**Fig. 2:**
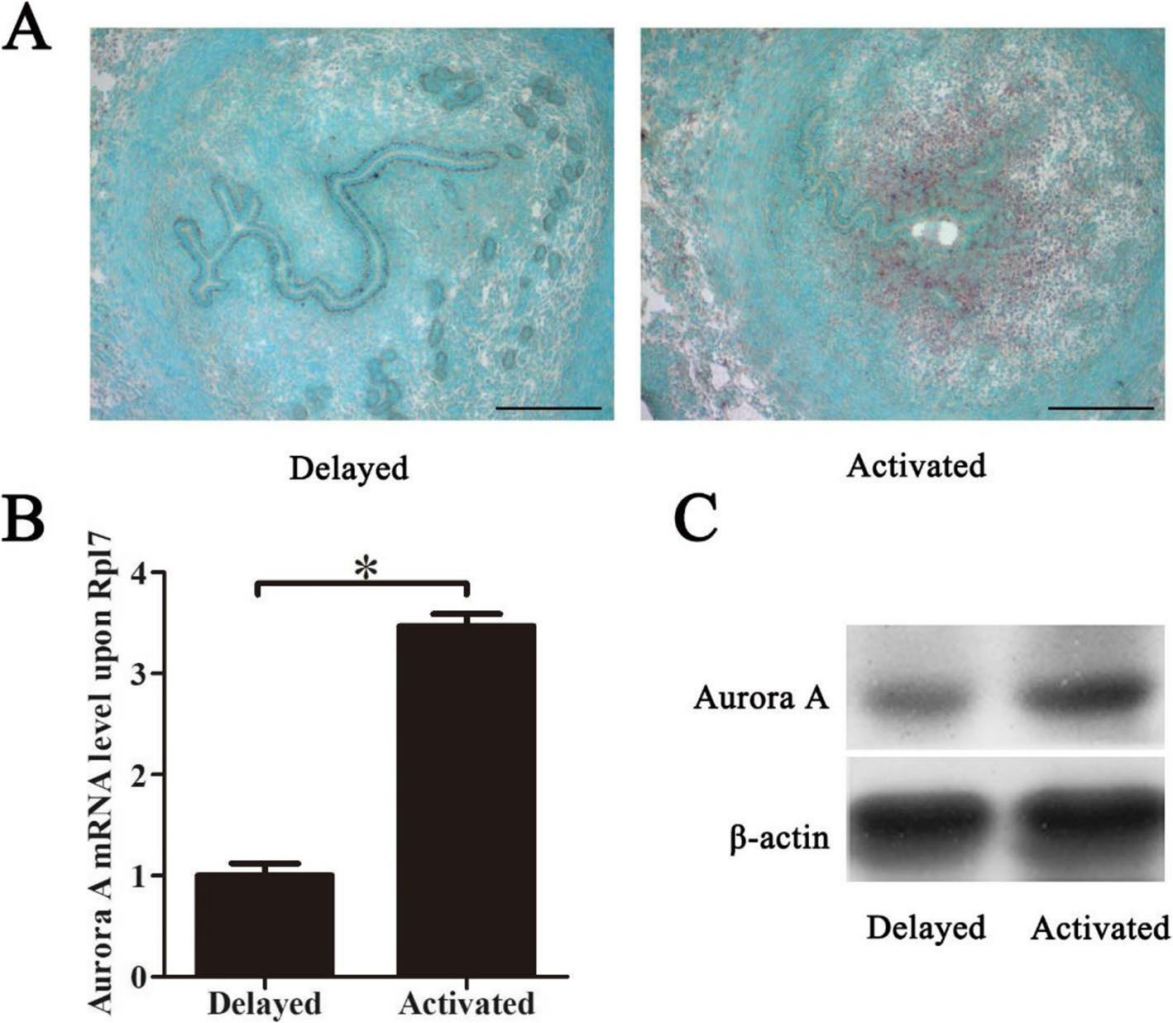
Aurora A expression in mouse uteri under delayed implantation. **A** In situ hybridization of Aurora A under delayed implantation. **B** Real-time PCR analysis of Aurora A expression under delayed implantation. **C** Western blot analysis of Aurora A protein. β-actin was used as a loading control. Data are presented as the mean ± SD, *p < 0.05, bar, 300 μm

To further check whether the expression of Aurora A is induced by the presence of an active blastocyst, a delayed implantation model was used. Under delayed implantation, there was no detectable Aurora A expression in mouse uterus (Fig. 2A). After delayed implantation was activated by estrogen treatment, Aurora A expression was highly detected in the stromal cells surrounding the implanting embryo (Fig. 2A). Based on real time PCR analysis, Aurora A expression was also significantly up-regulated in activated group compared with delayed group (Fig. 2B). Moreover, Aurora A protein level was stronger in activated group compared with delayed group by Western blot (Fig. 2C).

### Aurora A expression under artificial decidualization

**Fig. 3:**
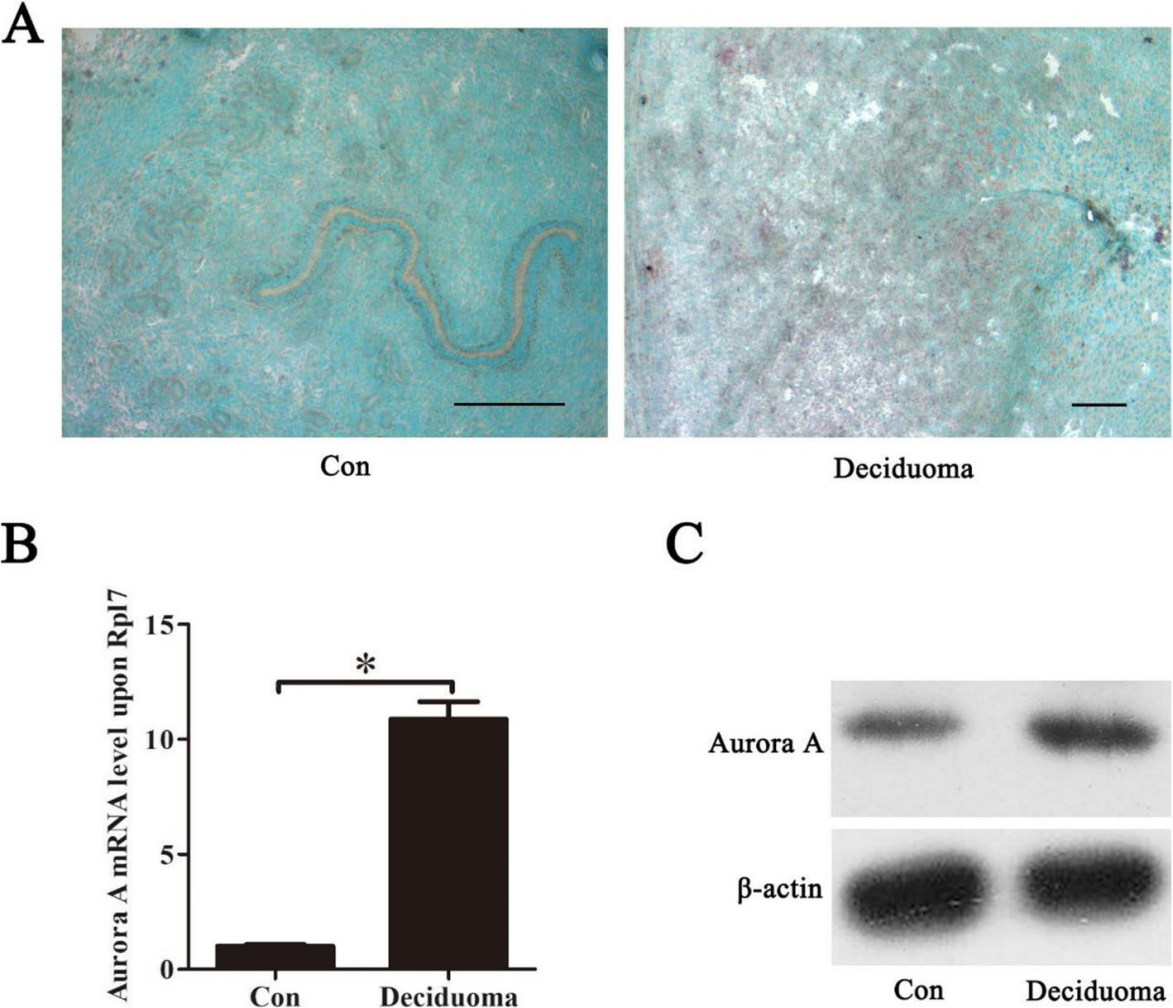
Aurora A expression in mouse uteri under artificial decidualization. **A** In situ hybridization of Aurora A expression under artificial decidualization. **B** Real-time PCR analysis of Aurora A expression under artificial decidualization. **C** Western blot analysis of Aurora A protein under artificial decidualization. β-actin was used as a loading control. Data are presented as the mean ± SD, *p < 0.05, bar, 300 μm

Because Aurora A was strongly expressed in the decidua during early pregnancy, artificial decidualization was performed to examine whether Aurora A expression was dependent on embryos. In the uninjected uterine horn, there was no detectable Aurora A mRNA signal (Fig. 3A). In the oil-induced uterine horn for artificial decidualization, Aurora A mRNA expression was observed in the decidualized cells (Fig. 3A). Results from real-time RT-PCR indicated that the level of Aurora A mRNA expression was significantly higher in the deciduoma compared with control (Fig. 3B). By Western blot analysis, the level of Aurora A protein in deciduoma was also higher than that in control group (Fig. 3C).

### Aurora A role during decidualization

**Fig. 4:**
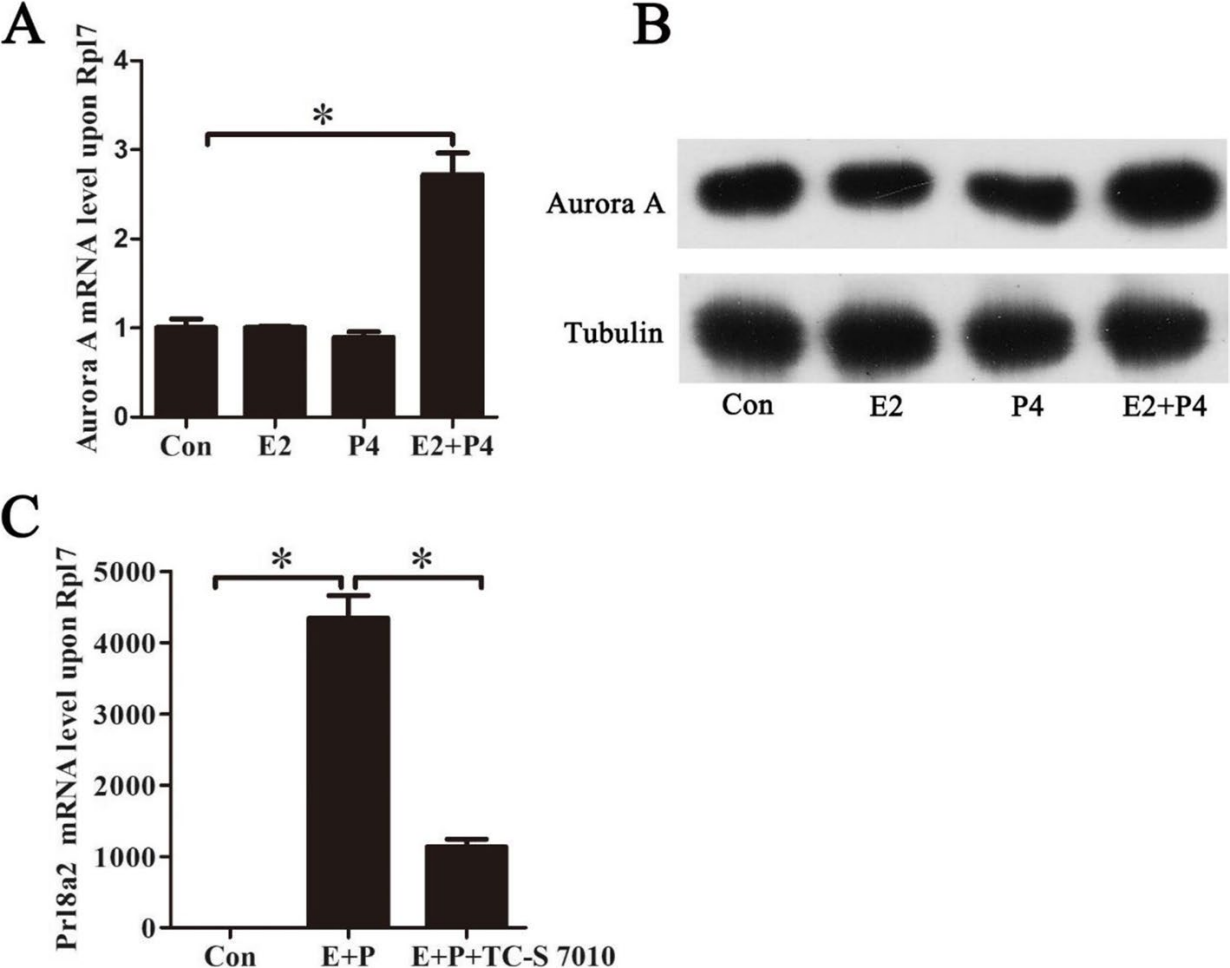
Aurora A expression under in vitro decidualization. **A** Real-time PCR analysis of Aurora A expression under in vitro decidualization (E2, Estradiol-17 β; P4, progesterone). **B** Western blot analysis of Aurora A protein level under in vitro decidualization. **C** Effects of TC-S 7010 on Prl8a2 mRNA levels. Tubulin was used as a loading control. Data are presented as the mean ± SD, *p < 0.05

Because Aurora A was strongly expressed in decidual cells from days 5 to 8 of pregnancy, in vitro decidualization model was used to examine whether Aurora A is involved in mouse decidualization. Compared to control, Aurora A expression was significantly induced under in vitro decidualization (Fig. 4A). Under in vitro decidualization, Aurora A protein level was also significantly upregulated compared to control (Fig. 4B). However, neither estrogen nor progesterone had detectable effects on the levels of Aurora A mRNA and protein (Fig. 4A, B). Prolactin family 8, subfamily A, member 2 (Prl8a2, previously named as Dtprp) is a reliable marker for mouse in vitro decidualization [28]. Under in vitro decidualization, Prl8a2 mRNA expression was significantly suppressed by TC-S 7010, a specific inhibitor for Aurora A (Fig. 4C). These results suggested that Aurora A might play a role during decidualization.

### Inhibition of Aurora A compromises mouse decidualization

**Fig. 5:**
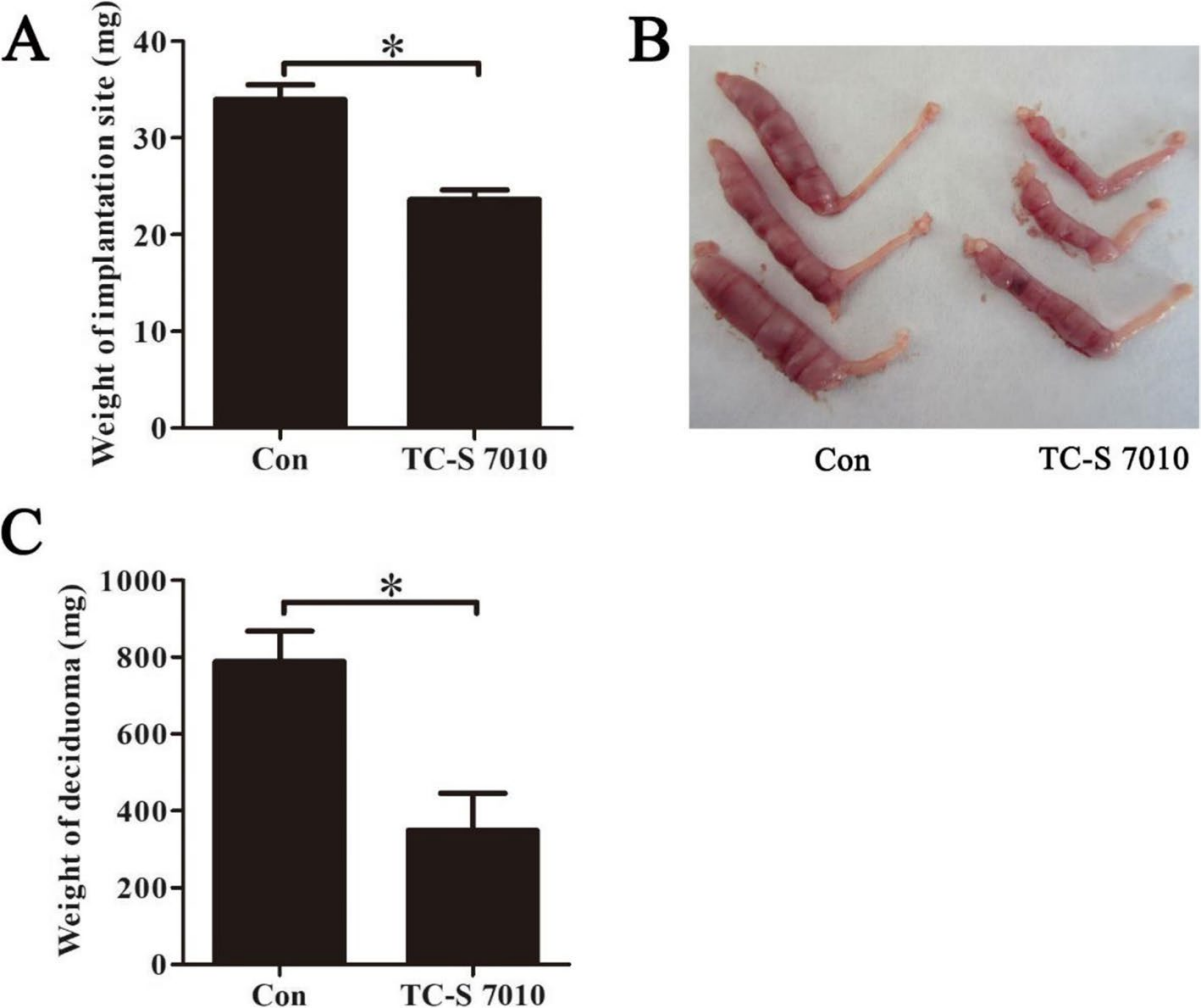
Effects of TC-S 7010 on decidua and deciduoma. **A** The weight of implantation sites after pregnant mice were treated with TC-S 7010. **B** The morphology of deciduoma following TC-S 7010 treatment in pseudopregnant mice. **C** The quantification of deciduoma after pseudopregnant mice under artificial decidualization were treated with TC-S 7010. Data are presented as the mean ± SD, *p < 0.05

Based on the role of Aurora A during in vitro decidualization, we further explore the role of Aurora A in vivo. After pregnant mice were daily treated with 30 mg/kg TC-S 7010 on days 6 and 7, the weight of implantation sites on day 8 was significantly decreased compared with control (Fig. 5A). To exclude effects of TC-S 7010 on embryonic development, pseudopregnant mice under artificial decidualization were daily treated with TC-S 7010 on days 6 and 7. Compared with control, the weight of deciduoma was also significantly reduced (Fig. 5B and C). These results indicated that Aurora A should be important for mouse decidualization.

### Involvement of Stat3, Plk1 and Cdk1 on regulating Aurora A during decidualization

**Fig. 6:**
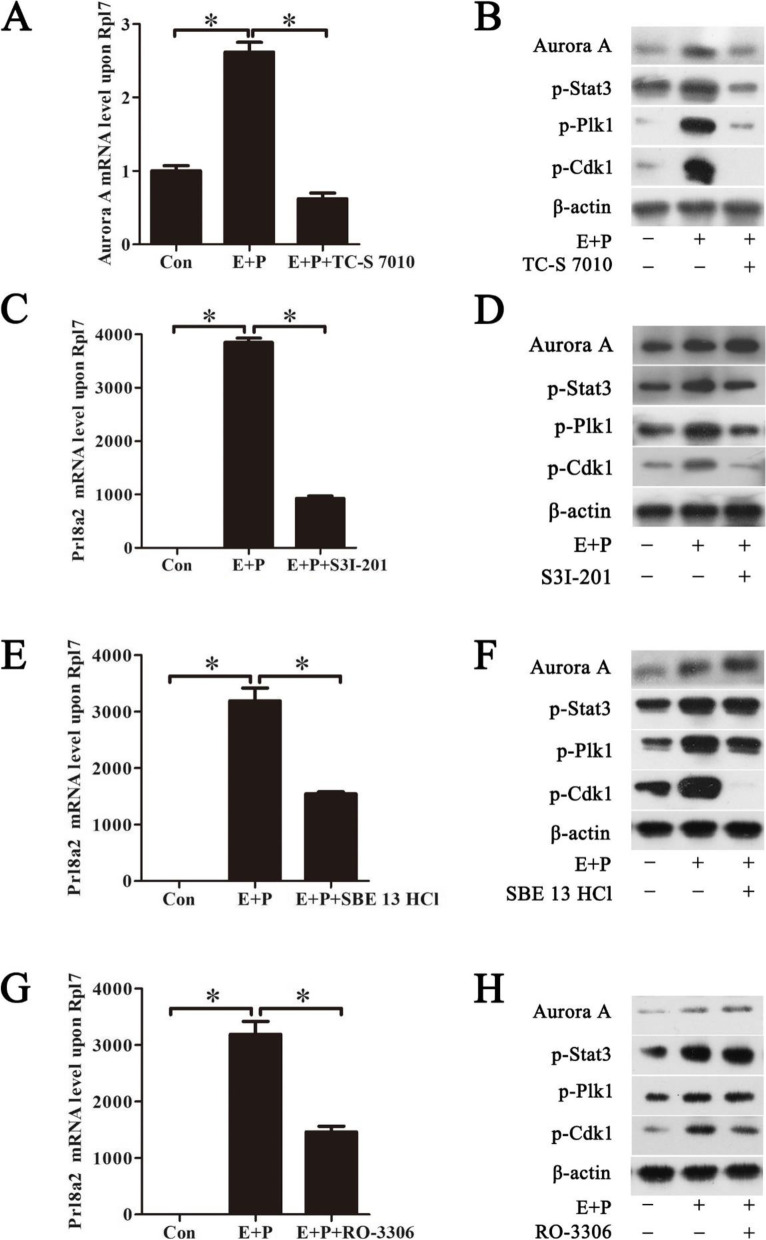
Effects of TC-S 7010, S3I-201, SBE 13 HCl and RO-3306 on in vitro decidualization. **A** Effect of TC-S 7010 on Aurora A mRNA level in stromal cells. **B** Western blot of Aurora A, p-Stat3, p-Plk1 and p-Cdk1 in stromal cells treated with TC-S 7010. **C** Effect of S3I-201 on Prl8a2 mRNA level in stromal cells. **D** Western blot of Aurora A, p-Stat3, p-Plk1 and p-Cdk1 in stromal cells treated with S3I-201. **E** Effect of SBE 13 HCl on Prl8a2 mRNA level in stromal cells. **F** Western blot of Aurora A, p-Stat3, p-Plk1 and p-Cdk1 in stromal cells treated with SBE 13 HCl. **G** Effect of RO-3306 on Prl8a2 mRNA level in stromal cells. **H** Western blot of Aurora A, p-Stat3, p-Plk1 and p-Cdk1 in stromal cells treated with RO-3306. β-actin was used as a loading control. Data are presented as the mean ± SD, *p < 0.05

Stat3 is strongly phosphorylated in luminal epithelium on day 4 of pregnancy and essential to blastocyst implantation in mouse uterus [12, 13, 15]. Plk1 promotes cell proliferation in renal cell carcinoma [29]. Cdk1 is strongly detected under mouse in vitro decidualization [24]. Moreover, Plk1 and Cdk1 are detected in human endometrium [30]. Therefore, we further explored whether p-Stat3, p-Plk1 and p-Cdk1 are involved in regulating Aurora A during mouse decidualization. Under in vitro decidualization, Aurora A mRNA expression was significantly decreased by TC-S 7010 (Fig. 6A). The protein levels of Aurora A, p-Stat3, p-Plk1 and p-Cdk1 were also significantly down-regulated by TC-S 7010 (Fig. 6B). After stromal cells were treated with Stat3 inhibitor (S3I-201) under in vitro decidualization, the expression of Prl8a2 mRNA was down-regulated by S3I-201 (Fig. 6C). S3I-201 treatment also suppressed the phosphorylation of Stat3, Plk1 and Cdk1, but had no effect on the expression of Aurora A (Fig. 6D), suggesting that Stat3 should be at the downstream of Aurora A. Under in vitro decidualization, SBE 13 HCl, a Plk1 inhibitor, significantly reduced Prl8a2 expression (Fig. 6E). Western blot showed that only p-Plk1 and p-Cdk1 were attenuated by the Plk1 inhibitor, but Aurora A and p-Stat3 levels weren’t decreased by Plk1 inhibitor (Fig. 6F), suggesting that Aurora A and Stat3 should be at the upstream of Plk1. Furthermore, RO-3306, a Cdk1 inhibitor, significantly inhibited Prl8a2 expression (Fig. 6G). Western blot indicated that p-Cdk1 was suppressed by RO-3306, but Aurora A, p-Stat3 and p-Plk1 levels weren’t inhibited by RO-3306 (Fig. 6H), suggesting that Cdk1 should be at the downstream of Aurora A, Stat3 and Plk1. These data indicated Aurora A-Stat3-Plk1-Cdk1 pathway was involved in mouse decidualization.

## Discussion

In this study, we showed that Aurora A is strongly expressed in the decidual cells at implantation sites. However, Aurora A expression is not detected at inter-implantation sites and under delayed uterus, suggesting that Aurora A expression is embryo dependent during peri-implantation period. Aurora A kinase expression is very low in the majority of endometriosis [31]. However, Aurora A kinase is only highly expressed in stromal and glandular cytoplasmatic component of malignant endometriotic lesions [31]. Therefore, Aurora A may be related to endometriosis and endometrial hyperplasia. Endometriosis reduces implantation rate and decidualization [32]. Dienogest is often used for hysteroscopic surgical treatment of endometriosis [33–35]. However, how Dienogest regulates Aurora A should be examined.

Mouse decidualization is triggered by the arrival of the blastocyst during pregnancy [36]. However, in the absence of a conceptus, decidualization in humans is triggered by progesterone [36]. Aurora A is an important during early pregnancy [37]. DNA methylation and Aurora kinase pathway act synergistically to repress genes on the inactive X chromosome [38]. Aurora A-induced oncogenic m6A modification acts as a key regulator of DROSHA in breast cancer [39]. Methylation of Aurora kinase A regulates cell proliferation and apoptosis [40]. Besides, aberrantly methylated Aurora A may be potential therapeutic targets for hepatitis C virus-positive hepatocellular carcinoma [10]. Aurora A is regulated by miR-137 in multiple myeloma [41]. Aurora A induces epithelial-mesenchymal transition through histone methylation in gastric cancer [42]. Mesenchymal-epithelial transition also occurs during decidualization. Current data showed that histone modification, DNA methylation and non-coding RNAs play roles during decidualization [43, 44]. Therefore, epigenetic modifications of Aurora A might be involved in during decidualization.

Aurora A expression is also detected in decidual cells under artificial decidualization. Aurora A is expressed in both normal and neoplastic endometria [45]. Uterine stromal cells extensively proliferate and differentiate into decidual cells at implantation site during decidualization [46]. As an cell cycle regulator, Aurora A may be involved in cell proliferation and differentiation during decidualization. Under in vitro decidualization, inhibition of Aurora A expression by TC-S 7010 leads to a notably reduction of Prl8a2, which is reliable marker for mouse in vitro decidualization. In our study, in vivo decidualization is also suppressed by TC-S 7010. Overexpression of Aurora A in cells enhances cell proliferation [47]. Furthermore, knocking down Aurora A by siRNA or specific inhibitor causes suppression of cell proliferation [26]. Aberrant Aurora A can lead to genomic instability and subsequently to arrest in mitosis [48]. Because Aurora A deficient mice die early during embryonic development before the 16-cell stage, it is unknown whether Aurora A is involved in early pregnancy [11]. Our data suggest that Aurora A is essential to mouse decidualization.

The occurrence of polyploid cells in decidua is a unique feature in rodents [49]. After embryos implant, polyploid decidual cells are detected in decidua and increase from days 5 to 8 of pregnancy [49]. The loss of death-effector domain-containing protein in mice results in attenuation of decidual polyploidy with seriously compromised decidualization, implicating polyploidy development is critically involved with decidualization [50]. Downregulation of Aurora A by special inhibitor SNS-314 promotes the polyploidy formation and apoptosis of hepatocellular carcinoma cells [51]. In mammary epithelial cells, increased expression of Aurora kinase-A and Polo-like kinase-1 at the lactogenic switch likely mediates the formation of binucleated cells [52]. However, in skin and mammary gland tumors, Aurora-A-deficient tumors accumulate polyploid cells, and conditional genetic ablation of Aurora-A results in polyploid cells [53]. Stat3 and Cdk1 are also involved in polyploid formation [24, 54]. MLN8237 (Alisertib), a selective inhibitor of AURKA, induced polyploidization and expression of mature megakaryocyte markers in acute megakaryocytic leukemia [55]. Therefore, we speculate that Aurora A should be involved in decidual polyploidy formation.

In this study, we demonstrated the effect of TC-S 7010 on Stat3 phosphorylation in stromal cells. It was previously reported to acquire enlarged polyploid nuclei and block Stat3 phosphorylation by inhibiting Aurora A in various cancer cell lines [56, 57]. Aurora A enhances Stat3 activity while Aurora A knockdown reduces the Stat3 nuclear translocation in gastric and esophageal cancers [16]. Furthermore, Stat3, a key signaling molecule in centrosome duplication [58], is involved in mouse decidualization and embryo implantation [12, 14]. Additionally, Stat3 may be involved in polyploidy formation caused by downregulation of endogenous MPHOSPH1 in hepatocellular carcinoma cells [59]. Our results showed p-Stat3 and Prl8a2 were blocked by TC-S 7010 and S3I-201 in mouse stromal cells. As master regulators in cell proliferation, Cdk1 and Plk1 are the key components in the control of the G2/M transition and contributes to mitotic progression [30]. Plk1 is critical for the initiation of centrosome maturation [60]. Furthermore, Plk1 expression in ectopic endometria is higher than that in eutopic endometria [30]. Plk1-null embryos are also embryonic lethal [61]. Meanwhile, the ablation of Aurora A leads to pre-implantation lethality in mice [37, 62]. Therefore, Aurora-A and Plk1 are essential to early mouse embryonic development. Plk1 upregulation induces the attenuation of cell cycle checkpoint control, leading to disruption of hepatocyte polyploidization in Full-length HBx transgenic mice [63]. Plk1 is activated by Aurora A in mitosis [20, 64]. As a downstream effector of Aurora A, Plk1 is vital for centrosome duplication process [65]. Cdk1 is indispensable for driving cell division in embryos development [66]. Moreover, Cdk1 is involved in decidual polyploidy formation [46, 67]. Aurora A is indispensable for recruitment of the cyclin B1-Cdk1 complex to centrosomes [68]. Our results showed that TC-S 7010 could suppress Plk1 and Cdk1, implying that Aurora A is most likely involved in regulating cell cycle progression. Cdk1 expression is inhibited in uterine stromal cells during mouse decidualization, which leads to G2/M phase arrest and polyploid stromal cells [24]. Moreover, Cdk1 is a central regulator that drives cells through G2 phase and mitosis [69]. As a maturation-promoting factor, cyclin B1/Cdk1 complex is responsible for regulating oocyte meiotic cell cycle progression [70]. Additionally, the repression of p-Plk1 and p-Cdk1 leads to cell polyploidy formation [71]. These results indicate that Aurora A may regulate the formation of polyploid decidual cells during mouse uterine decidualization via Stat3/plk1/cdk1 pathway.

## Conclusion

Our study shows that Aurora A is expressed in decidual cells and should be important for mouse decidualization. Aurora A/Stat3/Plk1/Cdk1 signaling pathway may be involved in polyploid decidual formation during mouse decidualization.

## Data Availability

All data generated or analyzed during this study are included in this published article.

## Abbreviations

Aurora A: Aurora kinase A
Prl8a2: Prolactin family 8, subfamily A, member 2
RO-3306: Cdk1 inhibitor
SBE 13 HCl: Plk1 inhibitor
S3I-201: Stat3 inhibitor
TC-S 7010: Aurora A inhibitor
